# Berberine suppresses tumorigenicity and growth of nasopharyngeal carcinoma cells by inhibiting STAT3 activation induced by tumor associated fibroblasts

**DOI:** 10.1186/1471-2407-13-619

**Published:** 2013-12-31

**Authors:** Chi Man Tsang, Yuk Chun Cheung, Vivian Wai-Yan Lui, Yim Ling Yip, Guitao Zhang, Victor Weitao Lin, Kenneth Chat-Pan Cheung, Yibin Feng, Sai Wah Tsao

**Affiliations:** 1 Department of Anatomy, The University of HongKong, HongKong, SAR, China; 2 Department of Otoloaryngology, University of Pittsburgh School of Medicine, Pittsburgh, USA; 3 Department of Anatomy, Histology and Embryology, Capital Medical University, Beijing, China; 4 School of Chinese Medicine, Li Ka Shing Faculty of Medicine, The University of HongKong, HongKong, SAR, China

## Abstract

**Background:**

*Cortidis rhizoma* (Huanglian) and its major therapeutic component, berberine, have drawn extensive attention in recent years for their anti-cancer properties. Growth inhibitory effects of berberine on multiple types of human cancer cells have been reported. Berberine inhibits invasion, induces cell cycle arrest and apoptosis in human cancer cells. The anti-inflammatory property of berberine, involving inhibition of **S**ignal **T**ransducer and **A**ctivator of **T**ranscription 3 (STAT3) activation, has also been documented.

**Methods:**

In this study, we have examined the effects of berberine on tumorigenicity and growth of nasopharyngeal carcinoma (NPC) cells and their relationship to STAT3 signaling using both *in vivo* and *in vitro* models.

**Results:**

Berberine effectively inhibited the tumorigenicity and growth of an EBV-positive NPC cell line (C666-1) in athymic nude mice. Inhibition of tumorigenic growth of NPC cells *in vivo* was correlated with effective inhibition of STAT3 activation in NPC cells inside the tumor xenografts grown in nude mice. *In vitro*, berberine inhibited both constitutive and IL-6-induced STAT3 activation in NPC cells. Inhibition of STAT3 activation by berberine induced growth inhibition and apoptotic response in NPC cells. Tumor-associated fibroblasts were found to secret IL-6 and the conditioned medium harvested from the fibroblasts also induced STAT3 activation in NPC cells. Furthermore, STAT3 activation by conditioned medium of tumor-associated fibroblasts could be blocked by berberine or antibodies against IL-6 and IL-6R.

**Conclusions:**

Our observation that berberine effectively inhibited activation of STAT3 induced by tumor-associated fibroblasts suggests a role of berberine in modulating the effects of tumor stroma on the growth of NPC cells. The effective inhibition of STAT3 activation in NPC cells by berberine supports its potential use in the treatment of NPC.

## Background

Traditional Chinese medicine represents a rich reservoir of potential small chemical molecules exhibiting anti-cancer properties [1]. Various natural products isolated from medicinal plants and their derivatives such as vinca alkaloid, etoposide, paclitaxel etc., are currently used successfully in cancer treatment [1,2]. The growth inhibitory effects of berberine have been recently reported in several types of human cancer cells, including hepatocelluar carcinoma, lung adenocarcinoma and breast cancer [3-5]. Berberine is an isoquinoline alkaloid and belongs to the structural class of protoberberines [6]. It is present in the roots, rhizome, and stem bark of a number of important medicinal plant species including *Cortidis rhizoma* (Huanglian)*, Berberis vulgaris* (barberry), *Coptis chinensis* (Chinese goldthread), and *Scutellaria baicalensis* (Baikal Skullcap), all of which have been used as traditional or folk medicines for centuries in China, India, Brazil and Peru [6,7]. Berberine is able to inhibit the growth of various types of cancer cells by inhibiting DNA topoisomerase I, inducing cell-cycle arrest and apoptosis through Fas/FasL signaling pathways and activation of caspase-3 [7]. In addition to their prominent anti-cancer activities, Berberine also exerts anti-inflammatory activities and inhibitory effects on growth and reproduction of tumorigenic microorganisms and viruses, such as *Helicobacter pylori* and hepatitis B virus [6,8]. We have previously reported that berberine can suppress the invasive properties of nasopharyngeal carcinoma (NPC) cell lines through inhibiting the activities of Rho GTPases [9]. Previous studies have also reported that berberine can suppress metastasis by enhancing the expression of a metastasis suppression gene, NM23-H1, or by targeting Rho kinase-mediated ezrin phosphorylation in NPC 5-8 F cell line [10,11]. In another study, we reported that berberine induces autophagic cell death and mitochondrial apoptosis in liver cancer cells [12]. Effective application of berberine as combined medication for tumor treatment has been reported [13,14]. Synergistic anti-tumor effects were also observed when berberine and irradiation were used in combination to treat lung cancer in both *in vivo* and *in vitro* models [14]. Another study indicated that berberine could enhance the anti-cancer effects of estrogen receptor antagonists on human breast cancer cells (MCF-7) through downregulating the expression of EGFR, HER2, Bcl-2, and COX-2, as well as upregulating IFN-α and p21 [13].

With this wide spectrum of anti-tumor properties, berberine has potential application as a complementary medicine for treatment and possibly prevention of human cancers. NPC is common among southern Chinese or Southeast Asian with an incidence rate of ∼ 30/100 000 per year in endemic regions such as Hong Kong and Guangzhou [15,16]. Besides its strong ethnic association with Southern Chinese, several epidemiological studies demonstrated that other risk factors are involved including Epstein-Barr virus infection, familial history, specific human leukocyte antigen (HLA) haplotype and male gender [16]. EBV infection is closely associated with undifferentiated type of NPC, which is the common histological type of NPC in southern Chinese, and has been postulated as an important etiological agent for NPC pathogenesis [16-18]. The majority of NPC patients (60–70%) are commonly presented with advanced diseases (Stages III and IV) at time of diagnosis. Despite the effective treatment by radiation and chemotherapeutic treatment, more than one third of NPC patients develop recurrence, some with distant metastasis [15].

Current research progress has revealed that the **S**ignal **T**ransducer and **A**ctivator of **T**ranscription 3 (STAT3) plays a pivotal role in NPC development [19]. Activation of STAT3 may contribute to both development and progression of NPC. STAT3-mediated oncogenesis can be attributed by the transcriptional upregulation of multiple downstream effector genes in cancer cells such as Mcl-1, which can promote cell growth, survival, and angiogenesis [20,21]. Our previous study also demonstrated a direct contribution of STAT3 activation to the invasive property of NPC cells [22]. STAT3 is activated in the majority of NPC patients (>75% of cases) and clinically correlated with advanced disease (stages III and IV) [23]. Thus, targeting aberrant STAT3 signaling may provide an effective and novel strategy for treatment of NPC [19].

Despite the fact that STAT3 activation is common in NPC, the mechanisms of STAT3 activation in NPC has not been fully elucidated. Cytokine-mediated STAT3 activation is believed to be a major mechanism driving STAT3 activation in several types of epithelial cancer [21]. As a matter of fact, development of NPC may be dependent on a highly inflammatory stroma. The tumor-infiltrating fibroblasts, macrophages, and lymphocytes release a myriad of inflammatory cytokines to support and maintain the growth and malignant properties of tumor [16]. Interleukin 6 (IL-6), a potent cytokine for STAT3 activation, was elevated in the sera of around 70% of NPC patients (out of 314 NPC patients) [24]. This elevation of serum IL-6 was also associated with the advanced diseases and the adverse prognosis of NPC. All these suggest that modulation of inflammatory responses in NPC by regulating the release of IL-6 and inhibition of STAT3 activation may suppress the development and growth of NPC.

Given the importance of STAT3 and inflammation in NPC pathogenesis, we set out to examine whether berberine could suppress activation of STAT signaling to exhibit anti-cancer effects using *in vitro* and *in vivo* models. Interestingly, we demonstrated for the first time that berberine could suppress the tumorigenic growth of NPC cells in athymic nude mice and inhibit the STAT3 activation. Berberine could also inhibit constitutive activation of STAT3 and its downstream effector, Mcl-1 in NPC cells. Furthermore, berberine also inhibited the activation of STAT3 by IL-6 in NPC cells. We also found that IL-6 secreted by tumor-associated fibroblasts could upregulate STAT3 activation in NPC cells in culture. By pre-treatment of the NPC cells with berberine, activation of STAT3 induced by tumor-derived fibroblasts was suppressed. Taken together, our results suggest that berberine may be a potential product from medicinal plant which can effectively inhibit the growth of NPC through suppression of STAT3 signaling.

## Methods

### Chemicals and antibodies

Berberine Chloride (C_20_H_18_ClNO_4_) was purchased from Sigma Chemicals (St. Louis, MO, USA). It was dissolved in sterile milli-Q water at a 80°C water bath for 10 mins to a stock concentration of 5 mM and stored at -70˚C before use. The primary antibodies used to detect p-STAT3 (Tyr 705), STAT3, cleaved-PARP-1, and β-actin were purchased from Cell Signaling Technology (Beverly, MA, USA). Antibody for detecting cytokeratin (clones AE1/AE3) was purchased from Dako (Carpinteria, CA, USA). The antibodies to detect Mcl-1, IL-6 (clone H-183) and the horseradish peroxidase (HPR)-linked secondary antibodies goat anti-mouse and goat anti-rabbit IgG were purchased from Santa Cruz Biotechnology (Santa Cruz, CA, USA). IL-6 and anti-IL-6-receptor antibody were purchased from R&D system (Minneapolis, MN, USA).

### Cell culture

C666-1 is a subclone of its parental cell line, C666, derived from an undifferentiated NPC xenograft of southern Chinese origin [25]. HONE-1 is derived from a poorly differentiated NPC from Chinese patient [26]. HK1 was established from a recurrent well-differentiated NPC of a Chinese patient after radiation therapy [27]. C666-1, HONE1 and HK1 cells were cultured in RPMI-1640 medium (Sigma) supplemented with 10% fetal bovine serum (FBS) (Sigma), 100 μg/ml penicillin/streptomycin (Invitrogen, Carlsbad, CA, USA) and maintained at 37˚C in a humidified atmosphere of 5% CO_2_. NP460 is an immortalized nasopharyngeal epithelial cell line established in our laboratory [28]. It is a non-tumorigenic cell line derived from normal nasopharyngeal tissue. It was cultured in a 1:1 mixture of Defined Keratinocyte-SFM (Invitrogen) and EpiLife™ medium with full supplements (Invitrogen). Tumour-associated fibroblasts were derived from primary cultures of NPC biopsies. Prior patient consents were obtained for the use of biopsied tissues for research investigation. The collection and use of the specimen have been approved by the Human Research Ethic Committee of the University of Hong Kong. The sample was collected in Queen Mary Hospital in Hong Kong. The NPC tissue was cut into small pieces (1 mm^3^) and left to grow in RPMI-1640 medium (Sigma) supplemented with 10% FBS (Sigma). After one week, the fibroblasts grew out from the NPC biopsies will be frozen down in liquid nitrogen for future use.

### *In vivo* nude mouse tumorigenicity assay

The *in vivo* nude mouse tumorigenicity assay was performed by injecting a total of 1 X 10^6^ C666-1 cells subcutaneously into the flank of 6–8 week old male nude mice. At the same day of injection of tumor cells, the drug was then administrated into the mice intraperitoneally (i.p.). The mice were either injected with saline (control group) or with berberine concentrations of 5 mg/kg body weight (low dose group) and 10 mg/kg body weight (high dose group) every other two days. Once tumor growth was established in the control mice (i.e. 14 days post-injection of tumor cells), tumor sizes were measured every other day. Tumor volume (mean ± SD) was calculated as length x width^2^/2 [29].

### Western blot analysis

Cell lysates were collected by scraping the cells with RIPA lysis buffer from the culture dishes, and the protein concentrations were determined using the DC Protein Assay Kit (Bio-Rad, Hercules, CA, USA) according to the manufacturer’s protocol. Equal amount of protein lysate (20 μg) per sample was resolved on 10% sodium dodecyl sulfate-polyacrylamide gel electrophoresis (SDS-PAGE) and transferred onto a polyvinylidene fluoride (PVDF) membrane (Amersham, Piscataway, NJ, USA). Membranes were probed with desired primary antibodies followed by detection of chemiluminescent signals of the peroxidase-conjugated secondary antibody using ECL Plus Western blotting detection system (Amersham, Buckinghamshire, UK). β-actin was used as an internal control to verify basal expression levels and equal protein loading. The ratio of the specific proteins to β-actin was calculated.

### Immunohistochemistry

Subcutaneous tumors developed in nude mice were excised on 43 days post-injection of C666-1 cells. Sections for immunohistochemistry were dewaxed in xylene and rehydrated in graded alcohol. Endogenous peroxidase activity was blocked by incubating slides with 3% hydrogenous peroxide for 10 min. For antigen retrieval, all slides were incubated with 10 mmol/L citrate buffer (pH 6.0) for 93°C for 10 min, and then cooled down to room temperature. After that, the sections were rinsed with PBS and treated with normal blocking serum (Vector Laboratories, Inc., Burlingame, CA, USA) for 30 min. Anti-p-STAT3 (1:100, Cell signaling) and anti-cytokeratin antibodies (1:200, Dako) diluted in PBS were applied to the sections and incubated at 4°C overnight. After rinsing, all sections were further incubated for 1 hr with biotin-conjugated secondary antibody and horseradish peroxidase-conjugated streptavidin followed by using diaminobenzidine (Dako) as a chromogen. Counterstaining was performed by hematoxylin before dehydration and mounting.

### ELISA for IL-6

The concentrations of IL-6 secreted from the NPC fibroblasts were quantitated using ELISA assay for IL-6 (R&D Systems) according to manufacturer instructions. Triplicated samples were estimated and the averages were used in the analysis.

### MTT assay

The effect of berberine on cell viability/ proliferation was determined using the MTT (3-[4,5-dimethylthiazol-2-yl]-2,5-diphenyl tetrazolium bromide) assay. MTT, a tetrazolium salt, is reduced to a purple blue formazan product by dehydrogenases in the mitochondria of living cells. The cell viability can thus be determined by quantifying the purple blue formazan product based on UV absorbance. Briefly, 5000 cells/well were plated in 96-well plates and incubated for 24 h. The cells were then treated with berberine at indicated concentrations for 24, 48 and 72 hr. The cells were then treated with 10 μl of 5 mg/ml MTT (Sigma) and incubated for 4 hr at 37˚C. The medium was then discarded, and 200 μl of dimethyl sulfoxide (DMSO) (Sigma-Aldrich, MO, USA) was added to dissolve the resulting formazan crystals. The absorbance was measured at 570 nm by the Multiskan MS microplate reader (Labsystems, Finland) with a blank reference at 650 nm.

### Statistical analysis

The data from each experiment were expressed as mean ± standard deviation (SD). Student’s *t* test was used to assess the differences between experimental groups. A *p* value < 0.05 was considered as statistically significant throughout this study.

### Ethical approval

The use of animals in this study was approved by the Committee on the use of live animals in teaching and research, the University of Hong Kong, Hong Kong.

## Results

### Berberine suppressed tumorigenicity and growth of NPC cells in nude mice

**Figure 1:**
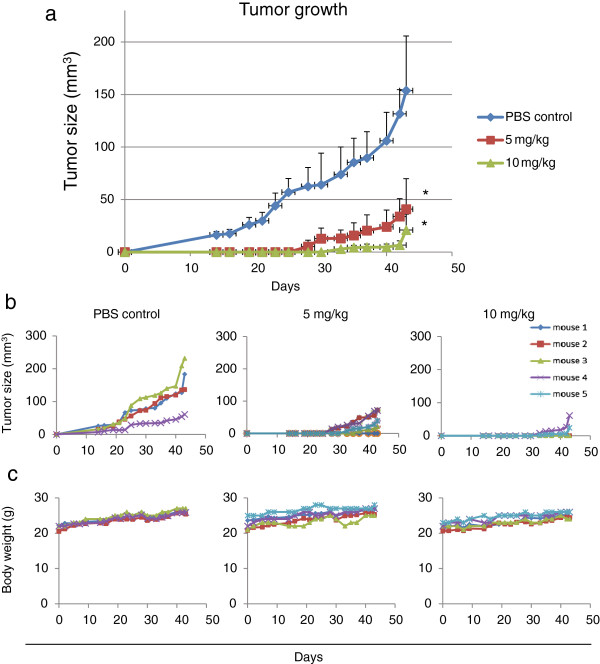
**Suppression of tumorigenicity and growth of NPC cells by berberine *in vivo*. (a)** Berberine could suppress the tumor initiation and growth of C666-1 xenografts in mice models. 1 X 10^6^ Tumor cells were injected subcutaneously into nude mice. Mice were either treated with berberine (low dose: 5 mg/kg or high dose: 10 mg/kg; i.p.) or treated with saline (as control) for every other two days. Both low and high dose of berberine could significantly suppress the tumor growth (*p* < 0.05) when compared to that of the control mice. **(b)** Individual growth curves of tumors in control and berberine-treated mice. The tumor sizes of individual mouse under control treatment (PBS-treated; n = 4), under low dose of berberine treatment (5 mg/kg berberine-treated; n = 5) and under high dose of berberine treatment (10 mg/kg berberine-treated; n = 5) were followed from 14 to 43 days post-injection of tumor cells. **(c)** The body weights of the mice within the treatment and control groups were comparable. The weights of the mice were recorded from the day of injection until 43 days post-infection of tumor cells. Both the control mice and berberine-treated mice showed a steady increase of body weight. Both 5 mg/kg and 10 mg/kg treatment of berberine did not cause drastic variation of body weight to the mice.

*Corptis rhizoma* is commonly used in several treatment remedies for cancer and berberine is the major active ingredient extracted from it [6,7]. *In vitro* inhibition of growth of human cancer cells by berberine has been reported by multiple studies [7]. It is crucial to examine any anti-tumor effects of natural product using *in vivo* tumor models. In this study, we have first assessed whether berberine could suppress the tumorigenic growth of NPC cells in immune deficient mice. The C666-1 cell line used for *in vivo* investigation harbours EBV infection which is representative of NPC from southern Chinese. To examine for inhibition of tumorigenicity and growth *in vivo*, 1 X 10^6^ C666-1 cells were injected subcutaneously into the flank of nude mice. On the same day of injection, the mice were either injected intraperitoneally (i.p.) with saline or low or high doses of berberine (5 mg/ and 10 mg/kg respectively) every two days. In the control mice (saline-treated), tumors started to appear after 14 days post-injection, and substantial growth of the tumor masses could be observed (Figure 1a). However, no measurable tumor masses could be detected in the berberine-treated mice until 30 days after injection of tumor cells in the 5 mg/kg berberine treatment group and 43 days in the 10 mg/kg berberine treatment group respectively (Figure 1a). By examination of the growth curves of individual tumor developed in control and berberine-treated groups, we could clearly observe a steady increase of tumor size in 4 out of 4 mice in control group after 14 days post injection (Figure 1b). Significant inhibition of *in vivo* tumorigenic growth of C666-1 was observed in both berberine treatment groups (5 mg/kg and 10 mg/kg). In treatment group injected with berberine (5 mg/kg), tumor growth was detected at 31 ± 5 days post-injection. In treatment group injected with higher dose (10 mg/kg), tumor growth was detected at 37 ± 5 days post infection (Figure 1b). Furthermore, tumorigenic growth of C666-1 was observed in only 2 out of 5 mice injected with 10 mg/kg (Figure 1b). These data suggest that berberine effectively suppresses the establishment and subsequent tumorigenic growth of NPC cells *in vivo*. Berberine is a natural compound found in traditional Chinese medicine and generally regarded as having low toxicity. Nonetheless, we have examined if there are any toxicity induced in berberine-treated mice in terms of mortality of the animals and alteration in body weight. The body weight and well-being of the control and berberine-treated mice were monitored periodically starting from day 1 of berberine administration. We did not observe significant alteration of body weight, morbidity and mortality after berberine treatment (Figure 1c). These results confirmed the findings from other reports stating that berberine is low in toxicity and does not induce adverse or detrimental effects on the general healthiness of mice.

### Berberine suppressed activation of STAT3 in NPC xenografts grown in nude mice

**Figure 2:**
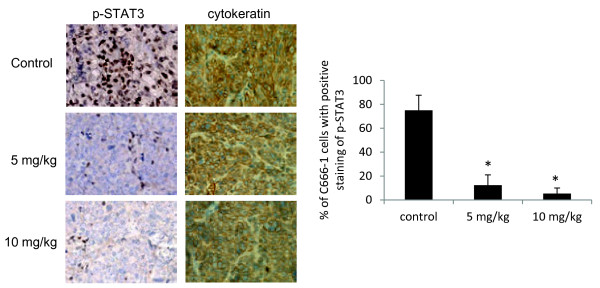
**Activation of STAT3 was inhibited by berberine *in vivo*.** Mice were continuously treated with saline (control group), low dose of berberine (5 mg/kg) and high dose of berberine (10 mg/kg) for every other two days up to a period of 43 days. The mice were then sacrificed and the tumors were sectioned and detected for p-STAT3. The sections were also stained with cytokeratin antibodies to identify the C666-1 cells from the fibroblasts. 5 random microscopic fields which consist of more than 95% of C666-1 cells were chosen for examination. Both low and high dose of berberine could significantly suppress the activation of STAT3 (*p* < 0.05) in C666-1 cells when compared to the controls.

Constitutive activation of STAT3 was commonly observed in NPC tissues and was known to promote growth, survival and progression of NPC [19,22,23]. In this study, we examined if berberine may suppress STAT3 activation in NPC grown as xenografted tumor in nude mice. The C666-1 NPC cells grown as subcutaneous tumor harvested from mice in control and treatment groups were embedded in paraffin, sectioned, and examined by immunohistochemical staining. Activated STAT3 will undergo phosphorylation and translocate to cell nucleus. Using antibody specific for phosphorylated STAT3 (p-STAT3), activated STAT3 could be demonstrated as brown nuclei stain in the tumor cells (Figure 2). We have also performed staining using antibody against cytokeratin to identify the C666-1 cells (epithelial in origin) from the stromal cells. For each section of tumors harvested from control and treatment groups, 5 random microscopic fields which contain over 95% of C666-1 cells were chosen for examination. The percentages of STAT3-activated C666-1 cells were counted. The tumor sections in the control group revealed 75% ± 12.6 of positive staining of p-STAT3 while only around 12.4 ± 8.6 and 5.4 ± 4.5% of cells were stained positive for p-STAT3 in the treatment groups injected with 5 mg/kg and 10 mg/kg of berberine respectively (*p* <0.05). Representative images showing the effective downregulation of p-STAT3 in the xenografted tumors treated with 5 mg/kg and 10 mg/kg of berberine are shown (Figure 2).

### Berberine suppressed the STAT3 signaling in NPC cell line *in vitro*

**Figure 3:**
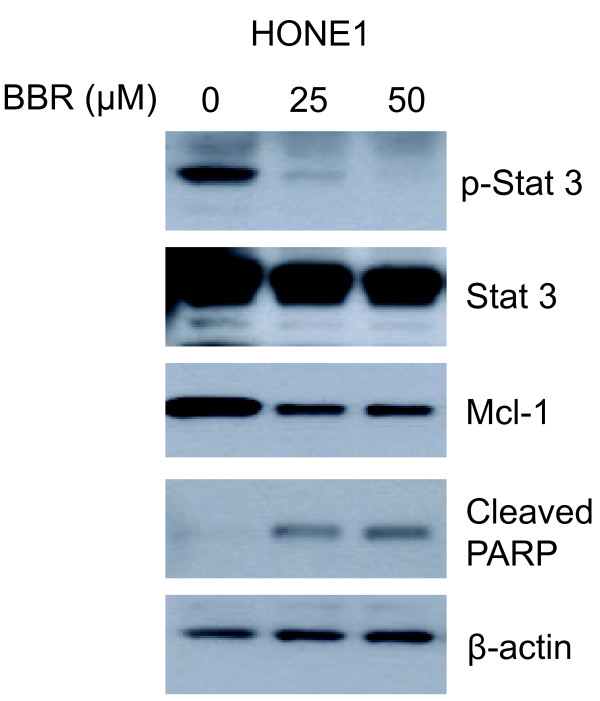
**Constitutive activation of STAT3 was downregulated by berberine in HONE1 cells.** HONE1 cells were treated with 25 and 50 μM berberine for 24 h. The activation of STAT3 was strongly suppressed by the treatment of berberine with a downregulation of survival protein (Mcl-1) and upregulation of apoptotic protein (cleaved-PARP-1). β-actin was probed for loading controls.

We then examined if berberine could suppress STAT3 in NPC cell grown *in vitro*. We first examined STAT3 activation in C666-1. Interestingly, STAT3 activation was not prominent in C666-1 grown in culture despite of its strong activation when grown *in vivo*. We postulated that STAT3 activation in C666-1 grown as xenograft may be induced by inflammatory signals from the host stroma. We have also investigated the effects of berberine on HONE1 which is an NPC cell line with high level of constitutive activation of STAT3 (Figure 3). High expression of p-STAT3 was detected in HONE1 cells. We observed that berberine could effectively suppress the level of p-STAT3 in HONE1 cells (Figure 3). Furthermore, the downregulation of STAT3 activation was associated with the suppressed expression of Mcl-1 (a downstream survival protein of STAT3) and an increased level of cleaved PARP-1 (an apoptotic marker) (Figure 3). This indicates that berberine could suppress constitutive activation of STAT3 in NPC cells and inhibited their survival ability by activating apoptosis.

### IL-6-mediated STAT3 activation in NPC cells was inhibited by berberine

**Figure 4:**
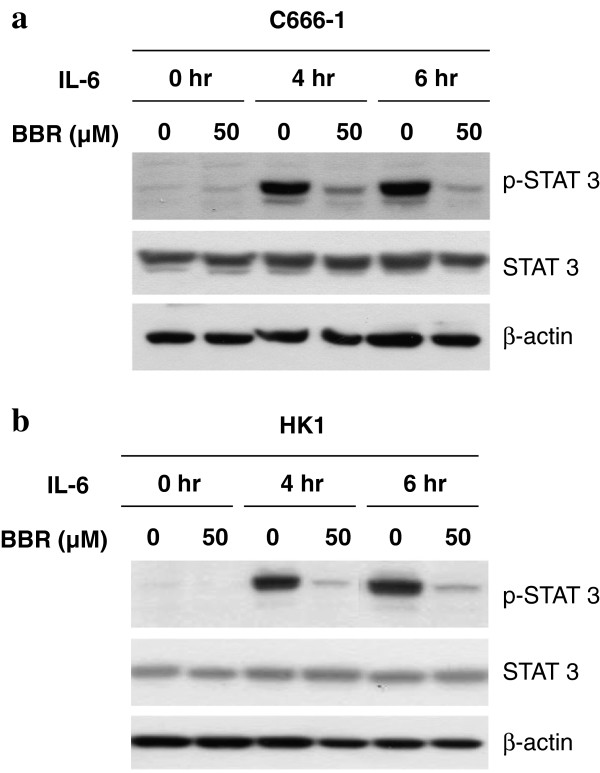
**Berberine inhibited the IL-6-activated STAT3 in C666-1 and HK1 cells.** Western blots analysis of **(a)** C666-1 and **(b)** HK1 cells with or without addition of berberine (50 μM) after IL-6 treatment (20 ng/ml) for 0, 4 and 6 hr. The cell lysate was probed with anti-STAT3 and anti-p-STAT3. Equal protein loading was established with anti-β-actin antibody.

Next, we examined if activation of STAT3 by extracellular stimulus, such as IL-6, could also be suppressed by berberine. IL-6 is one of the major inflammatory cytokine present in NPC tissues. It has been reported that there is a positive feed-back loop of IL-6/STAT3 signaling in NPC to potentiate STAT3 signaling which enhances malignant properties of NPC [30]. In this study, IL-6 (20 ng/ml) was added to two NPC cell lines (C666-1 and HK1), which have low basal level of STAT3 activation, for 0, 4 and 6 hr in the presence or absence of 50 μM of berberine. Figure 4 shows that expression of p-STAT3 in both cell lines could be induced by IL-6 in a time-dependent manner. Berberine could effectively suppress the IL-6-induced p-STAT3 in both NPC cell lines (Figure 4).

### Berberine exhibited low toxicity towards cell lines with low basal activation of STAT3

**Figure 5:**
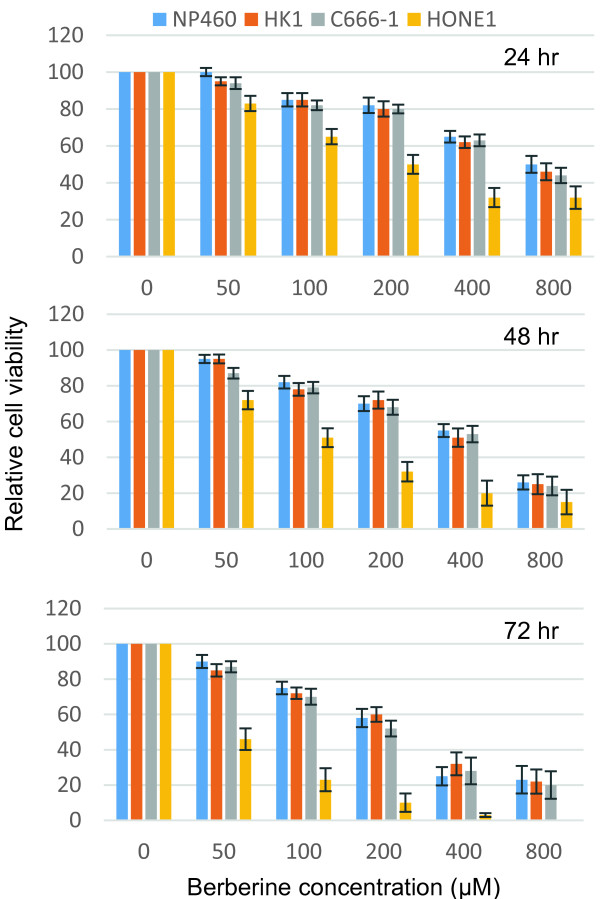
**Berberine exhibited higher toxicity towards HONE1 cells.** MTT assays were performed on one non-neoplastic nasopharyngeal epithelial cell line (NP460), and three NPC cell lines (HK1, C666-1 and HONE1) for 24, 48 and 72 hr under the indicated doses of berberine (0, 50, 100, 200, 400 and 800 μM). For each time point, the growth rates of each untreated cell line were arbitrarily assigned as 100. Mean ± SD were calculated from triplicate of two independent experiments.

We have previously demonstrated that berberine could potently suppress the IL-6-induced or constitutive activated STAT3 in NPC cells. We speculated that berberine may be able to target cancer cells which are dependent on STAT3 activation for growth and survival. We performed MTT assays to assess the cytotoxicity of berberine on three NPC cell lines (HK1, C666-1 and HONE1) and also one normal immortalized nasopharyngeal epithelial cell line (NP460) . Interestingly, berberine had high toxicity towards the HONE1 cell line, which has constitutively activated STAT3 (Figure 5). The IC50 after 24 hr berberine treatment for HONE1 was around 100 μM, while the IC50 after similar treatment for HK1, C666-1 and NP460 was around 400 μM (Figure 5).

### STAT3 activation stimulated by fibroblast-secreted-IL-6 could be inhibited by berberine

**Figure 6:**
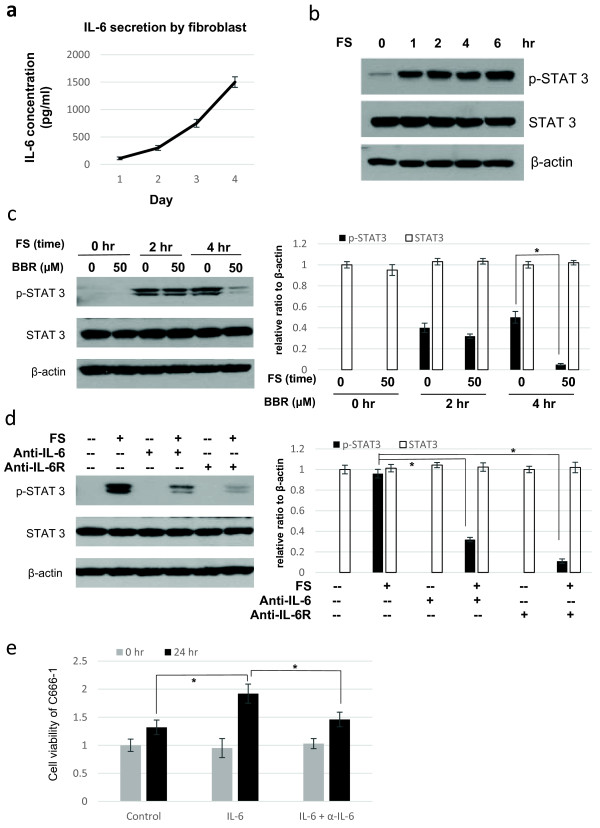
**Berberine could suppress the STAT3 activation in NPC cells induced by IL-6 secreted from fibroblasts. (a)** IL-6 was secreted by tumor-associated fibroblasts. 1 x 10^6^ fibroblasts were seeded in each well of a 6-well plate. Fibroblast supernatant was collected after 1, 2, 3 and 4 days of incubation in culture hood. The concentration of IL-6 was assessed by ELISA. **(b)** Fibroblast supernatant (FS) could activate the STAT3 signaling in C666-1 cells. FS collected after 4 days of incubation was used to treat C666-1 for 1, 2, 4 and 6 hr. Increasing levels of p-STAT3 could be detected in a time-dependent manner. **(c)** Berberine could inhibit the activation of STAT3 induced by FS. 50 μM of berberine could effectively suppress the FS-induced STAT3 activation at 4 hr time points. Three independent experiments were carried out, relative ratio to β-actin was calculated, and data are represented as mean ± S.D. from three experiments. *, *p* < 0.05. **(d)** The activation of STAT3 in FS-treated NPC cells was dependent on the IL-6\IL-6R signaling pathway. C666-1 cells were pretreated with anti-IL-6 or anti-IL-6R antibodies for 30 mins before the addition of FS. Protein was collected after 4 hr treatment of FS. Anti-IL-6 or anti-IL-6R antibodies could significantly suppressed the FS-induced activation of STAT-3. Three independent experiments were carried out, relative ratio to β-actin was calculated, and data are represented as mean ± S.D. from three experiments. *, *p* < 0.05. **(e)** IL-6 could enhance the growth rate of C666-1 cells. The cells were treated with IL-6 (20 ng/ml) or pretreated with anti-IL-6 antibody for 30 mins before the addition of IL-6. After 24 hr, the growth rate was determined by MTT assay. Mean ± SD were calculated from triplicate of two independent experiments. *, *p* < 0.05.

*In vivo*, fibroblasts in tumor tissue have been postulated to serve as a source of pro-oncogenic cytokines, including IL-6 [31]. Here, we used fibroblasts isolated from primary human NPC tumor biopsy to develop an *in vitro* system to mimic the *in vivo* secretion of IL-6 by stromal cells and the subsequent activation of STAT3 in cancer cells. The primary fibroblasts derived from NPC tissue were cultured in serum free medium for 4 days and culture supernatants from day 1 to day 4 were collected for investigation. The concentrations of IL-6 in the culture supernatants were measured by ELISA, and was found to progressively increase to a concentration of around 1,500 pg/ml at day 4 (Figure 6a). This concentration of IL-6 was sufficient to stimulate STAT3 activation in C666-1 cells. STAT3 activation as indicated by the phosphorylation of STAT3 was induced at a time-dependent manner in C666-1 cells after incubation with fibroblast-conditioned medium (Figure 6b). The STAT3 activation in the NPC cells by fibroblast-conditioned medium could be suppressed by treating the cells with 50 μM berberine (Figure 6c). To further confirm that IL-6 in the fibroblast-conditioned medium was involved in the activation of STAT3 in C666-1 cells, anti-IL-6 and anti-IL-6-receptor antibodies were added to the fibroblast-conditioned medium. Both antibodies could effectively inhibit the activation of STAT3 (Figure 6d). It could be deduced that IL-6 secreted from fibroblasts play a key role in the activation of STAT3 in C666-1 cells and activation of IL-6-receptor on NPC cells was involved. Furthermore, IL-6 treatment also increased the growth rate of C666-1 cells (Figure 6e).

## Discussion

There are ample evidences supporting a functional role of STAT3 in tumorigensis and progression of NPC through promotion of tumor initiation, growth and invasive properties of cancer cells [19,22,32]. STAT3 activation emerges as a potential target for therapeutic treatment of NPC [19]. In this study, we found that berberine could effectively inhibit the growth of NPC xenografts in nude mice (Figure 1) and inhibition of STAT3 activation was likely to be involved (Figure 2). *In vitro* experiments provide evidences that the inhibitory action of berberine on NPC cells could be mediated by directly inhibiting the constitutive activation of STAT3 or by inhibiting the STAT3 activation induced by exogenous pro-inflammatory cytokines such as IL-6 (Figures 3, 4, 5, 6).

Several studies have shown that STAT3 is a potential target for anti-cancer therapy [19,21,33]. Treatment with STAT3 inhibitor selectively suppressed the growth, viability, survival and malignant transformation of human breast (MDA-MB-231) and pancreatic (Panc-1) cancer lines, and down-regulated the expression of known STAT3-regulated genes, including c-Myc, Bcl.xL, the matrix metalloproteinase 9, and VEGF [34]. It could induce strong tumor regression in xenografts of the human breast cancer [35]. Furthermore, recent evidences indicate that STAT3, apart from being a target for anti-cancer therapy, may also represent a crucial target for cancer prevention as STAT3 plays an essential role in tumor formation and initiation [32,35]. STAT3-deficient mice were completely resistant to skin tumor development in a chemical-induced skin tumorigenesis model [36]. Furthermore, in breast cancer, abrogation of STAT3 activation inhibited tumor formation in the mammary fat pad of a syngeneic model [37]. In NPC, one of our previous publications also showed that brief exposure of tumor cells with JAK/STAT3 inhibitor could efficiently suppress the tumor initiation in nude mice [32]. In this study, we demonstrate the suppressive effect of tumor growth by berberine through inhibiting STAT3 activation. Berberine has been administrated into human bodies for a history of thousands of years with few indications of serious adverse effects. In an early study, berberine was shown not to cause any histopathological changes in rat tissues and organs when administered for 6 weeks in 500 mg/kg daily oral doses [38]. We also observed that the berberine-treated mice did not suffer from loss of body weight when compared to PBS-treated mice (Figure 1c). The low toxicity of berberine and its effective inhibition of STAT3 and tumorigenic growth of NPC make it a potential anti-cancer drug or even chemopreventive agent for NPC through its inhibitory effects on inflammation which is common in premalignant and cancerous NPC tissues.

We have further examined the inhibitory effect of berberine against STAT3 activation using various NPC cell lines grown *in vitro*. One of our NPC cell lines, HONE1, is constitutively activated in STAT3 without the need of extracellular stimulus. Berberine was shown to effectively suppress the constitutive activation of STAT3 in HONE1 cells (Figure 3). The downstream effector of STAT3, Mcl-1 (a survival protein) was also significantly downregulated by berberine treatment which was accompanied by upregulation of cleaved-PARP-1 (apoptosis marker) (Figure 3).

The underlying reason for common STAT3 activation in NPC is not completely defined. EBV infection, and stimulation with cytokines from inflammatory stroma may activate STAT3 in NPC [19,39]. We have investigated if berberine could inhibit STAT3 activation in NPC cells by exogeneous stimulus, IL-6, which is commonly secreted by inflammatory stroma cells present in tumor microenvironment. Elevation of serum IL-6 was detected in more than 70% of NPC patients [24]. An earlier study has indicated the involvement of STAT3 in mediating the IL-6/LMP1 feedback in LMP-1-expressing cell or EBV-infected cells [30]. In our recent study, we reported long-term propagation of EBV infection in nasopharyngeal epithelial cells enhances IL-6-mediated STAT3 activation [39]. Hence, EBV infection in NPC may potentiate the activation of STAT3 in infected cells under an inflammatory stroma, in which IL-6 is highly expressed. In the present study, we found that both C666-1 (an EBV-infected NPC cell line) and HK1 (a non-infected NPC cell line) which have low basal level of STAT3 activation, were responsive to exogenous STAT3 activation by IL-6. Berberine was shown to potently suppress the IL-6-activation of STAT3 in both cell lines (Figure 4). By performing MTT assay on C666-1, HK1, HONE1 and immortalized NP460 cells, we found that berberine has low toxicity towards the cells with low basal levels of activated STAT3, but has higher toxicity towards HONE1 cell line, which has constitutively activated STAT3 (Figure 5). The much lower IC50 of HONE1 compared to the other cell lines may reflect its dependency for STAT3 activation for growth and survival. This also suggests that the suppressive effect of berberine on the tumorigenicity of C666-1 in nude mice might due to its inhibition on STAT3 activation when grown *in vivo.* To mimic the *in vivo* activation of STAT3 in tumor cells with cytokines secretion from stroma cells in tumor, we used the fibroblast-conditioned supernatant to induce STAT3 activation in NPC cells (Figure 6a and b). Berberine was able to suppress the STAT3 activation in NPC cells induced by fibroblast supernatant and inhibition of IL-6-induced STAT3 activation was involved (Figure 6c and d). At last, IL-6 could enhance the growth of NPC cells *in vitro* (Figure 6e). All these results support a novel role of berberine to inhibit STAT3 signaling in NPC by targeting fibroblasts present in tumor stroma. Besides, STAT3 mediated pro-oncogenic inflammation has also been well documented to promote tumor initiation and progression [40,41]. In view of the effective action of berberine to inhibit STAT3 and relatively low toxicity, berberine may serve as an effective chemopreventive agent or conjugant medicine for treatment of NPC through modulating the inflammatory tumor microenvironment.

## Conclusion

Berberine could suppress the growth and activation of STAT3 of NPC cells *in vivo*. It could also inhibit both the constitutive and IL-6-induced STAT3 activation *in vitro*. Higher cytotoxicity of berberine was exhibited in cell line with constitutive activation of STAT3. It suggests that berberine can suppress the growth and survival of NPC cells which are dependent on STAT3 activation for tumorigenicity. Besides, berberine could abrogate the activation of STAT3 in NPC cells induced by the IL-6 secreted from tumor-associated fibroblasts. All these support the potential use of berberine in the treatment of NPC.

## Competing interests

The authors declare that they have no competing interests.

## Authors’ contributions

TCM and CYC designed research; CYC, CKCP, TCM, YYL and ZG performed research; CYC and TCM analyzed data, TCM, LVW, FY and TSW wrote the paper. All authors read and approved the final manuscript.

## Pre-publication history

The pre-publication history for this paper can be accessed here:

http://www.biomedcentral.com/1471-2407/13/619/prepub
